# Enhancing the Delivery of Chemotherapeutics: Role of Biodegradable Polymeric Nanoparticles

**DOI:** 10.3390/molecules23092157

**Published:** 2018-08-27

**Authors:** Jyoti Ahlawat, Gabriela Henriquez, Mahesh Narayan

**Affiliations:** 1 The Department of Chemistry & Biochemistry, The University of Texas at El Paso, El Paso, TX 79968, USA; jahlawat@miners.utep.edu; 2 Environment Science & Engineering Department, The University of Texas at El Paso, El Paso, TX 79968, USA; ghenriquez@miners.utep.edu

**Keywords:** polymeric nanoparticles, mononuclear phagocyte system, drug delivery, chemotherapeutic drugs, drug toxicity

## Abstract

While pharmaceutical drugs have revolutionized human life, there are several features that limit their full potential. This review draws attention to some of the obstacles currently facing the use of chemotherapeutic drugs including low solubility, poor bioavailability and high drug dose. Overcoming these issues will further enhance the applicability and potential of current drugs. An emerging technology that is geared towards improving overall therapeutic efficiency resides in drug delivery systems including the use of polymeric nanoparticles which have found widespread use in cancer therapeutics. These polymeric nanoparticles can provide targeted drug delivery, increase the circulation time in the body, reduce the therapeutic indices with minimal side-effects, and accumulate in cells without activating the mononuclear phagocyte system (MPS). Given the inroads made in the field of nanodelivery systems for pharmaceutical applications, it is of interest to review and emphasize the importance of Polymeric nanocarrier system for drug delivery in chemotherapy.

## 1. Introduction

Nanotechnology refers to the study of materials that have at least one dimension in the nanometer range. Therefore, it would not be wrong to call it as the technology of future since it involves designing nanostructures through methodologies that involve control over physical and chemical characteristics at molecular level i.e., nanoscale [1]. Since the famous 1959 lecture “There’s Plenty of Room at the Bottom”, by the Nobel laureate Richard P. Feynman, there have been many developments in the field of biology, chemistry, and physics that demonstrate his idea of managing and operating matter at the level of molecules and atoms [2]. Related to this, a more accurate definition of nanotechnology was given by Drexler in 1981 which involves production of material with dimensions and precision between 1 and 100 nm [3]. Nanomaterials are one of the fastest emerging research areas and is an interdisciplinary field that requires understanding and expertise in diverse areas such as biology, chemistry, electronics, engineering, physics and social sciences. As the research on nanomaterials is growing, it would not be wrong to call this period of development in nanomaterials as Nano era. Furthermore, Gutierrez in 2005 reported the possibility of conversion of all structures into nanostructures. For instance, it has been more than a decade now since nanomaterials are used to improve the quality and grit of products [4,5]. Therefore, success of nanotechnology lies in influencing the purity and composition (physical and chemical) of the parent material [6].

**Figure 1:**
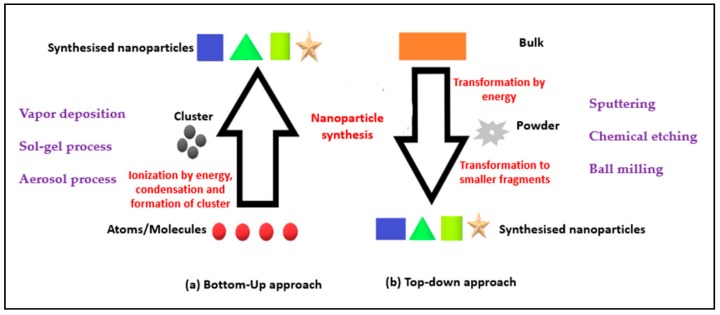
Schematic representation of (**a**) bottom-up; and (**b**) top-down approach.

Nanotechnology encompasses two approaches: (1) A top-down approach; and (2) a bottom-up approach (Figure 1):

1. *“Top-Down” approach:* The nanoscale structures are deconstructed from larger structures while maintaining their original properties and chemical composition [2,6].
2. *“Bottom-Up” approach:* Involves engineering the materials at atomic or molecular level through a process of assembly or self-assembly [2].

The chemical synthesis methods involved have influence on the size and shape of the nanoparticle constructed using the bottom-up approach. Although the contemporary methods rely more on the top-down approach, the bottom-up approach produces more ordered or crystalline nanoparticles resulting in a greater change in their surface energies and morphologies. Apart from the various applications of bottom-up approach in materials and manufacturing, electronics, medicine and healthcare, energy and biotechnology, to name a few, the limitations include its high operational cost, limited suitability (for laboratory use only) and expertise requirement [2,7].

In this review, we will focus on the use of polymeric nanoparticles for cancer treatment. Currently, more than 90% of the available potential therapeutics have poor pharmacokinetic properties. Therefore, there is an urgent and unmet need to produce drug delivery systems that can distribute drug molecules to the targeted site without affecting surrounding healthy cells [7]. In this context, nanoparticles possess several advantages, such as: Lower doses, improved pharmacokinetics, increased delivery to target site, reduced drug toxicity, reduced liver clearance, improved solubility of hydrophobic drug in aqueous medium and bio-availability, and increased stability of therapeutic agents such as peptides and oligonucleotides. Furthermore, biocompatibility of nanoparticles helps in avoiding hypersensitivity reactions and peripheral neuropathy and can be injected without occluding needles [7].

## 2. Types of Nanoparticles for Drug Delivery

**Figure 2:**
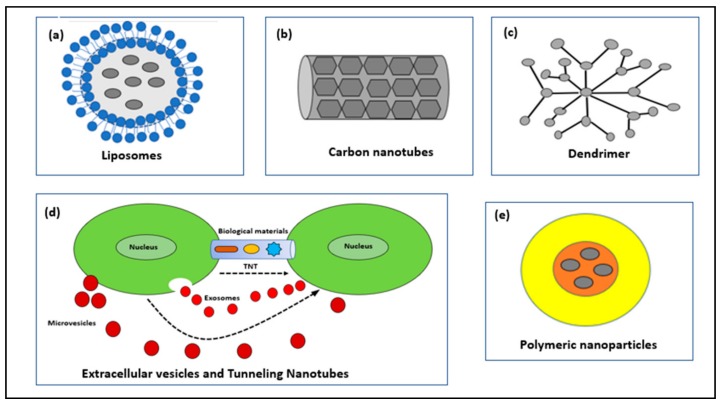
Schematic illustration of nanocarriers for the delivery of drug.

Nanoparticles used as drug conjugates can be classified into: Liposomes, Carbon Nanotubes, Dendrimers, Extracellular Vesicles, Tunneling Nanotubes and Polymeric nanoparticles (Figure 2).

### 2.1. Liposomes

Liposomes are bilayered vesicles composed of an outer lipid bilayer surrounding an inner aqueous core. The biocompatibility, amphiphilic nature, and ease of surface modification allow for increased circulation time. These properties enable the liposome to deliver the drug either by adhering to the cell membranes or by the process of endocytosis [8,9,10,11].

Due to their stability related issues, they have limited medical impact but are extensively used in cosmetic products. Moreover, functionalization using Polyethylene glycol (PEG) allows for enhanced circulation time. Liposomal formulations such as Doxil, Myocet and DaunoXome are approved for metastatic breast cancer treatment and Kaposi’s sarcoma [12,13,14].

### 2.2. Carbon Nanotubes (CNTs)

Nanotubes can be inorganic or organic (carbon nanotubes) in composition. Carbon nanotubes are self-assembling sheets of atoms arranged in tubes. For instance, they can have single or multi-walled structure with the latter one being more stable as the aggregation tendency decreases with reduced nanocurvature. Furthermore, the chemical modification of Carbon Nanotubes (CNTs) make them soluble and functionalized so that active substances like peptides or drugs can be attached on their surface [15]. They also have large internal volume. However, acute toxicity, carcinogenesis, immunogenicity arising due to the use of these nanoparticles cannot be overlooked. Therefore, they must be chemically or biologically modified before use for cellular delivery. Properties like low biocompatibility limit its use [16,17,18,19]. Although, they have been used as biosensors, drug delivery vehicle and as diagnostic tools, their insolubility in all solvents has caused health related issues.

### 2.3. Dendrimers

The term dendrimer was proposed due to its resemblance to a tree [20]. It is a synthetic polymer-based macromolecule in nanometer range having multiple branched monomers radiating out from the central core [21]. For instance, the void at the center, multivalence, ease of surface modification, well-defined globular-shape, predictable molecular weight, lack of immunogenicity, water solubility and size control make them a desirable candidate for drug delivery [21]; among these are PAMAM (polyamidoamine) and PPI (polypropylene). Although they can be conjugated with multiple substances, like imaging agent, drug, targeting ligand forming a multifunctional drug delivery system, biocompatibility and biodistribution problems limits their application [15,22].

### 2.4. Extracellular Vesicles (EVs) and Tunneling Nanotubes (TNTs)

These are basically lipid based bilayered structures composed mainly of cermaides, cholesterol and sphingolipids. Intracellular communication and cargo transfer via extracellular vesicles are well known and is gaining focus in research [23]. Furthermore, EVs is a generalized term and based on distinct biogenesis and release pathways these are further classified into various subtypes such as exosomes (50–100 nm), ectosomes (100–1000 nm) and oncosomes (1–10 um) [24]. For instance, these can be round, or cup shaped in morphology when observed under Scanning Electron Microscope (SEM). Moreover, these are well known for transfer of biological materials in paracrine fashion [23]. 

Rustom et al. in 2004, discovered an actin-based transient extension of cytoplasm between the cells, in the range of 50–200 nm, resembling nanotubes with open ends, called tunneling nanotubes [25]. The biological synthesis of this nanostructure is based on f-actin polymerization [26,27]. Furthermore, TNTs have role in intercellular communication, transfer of cargo, immunoregulation and inflammatory response [23].

### 2.5. Polymeric Nanoparticles

**Figure 3:**
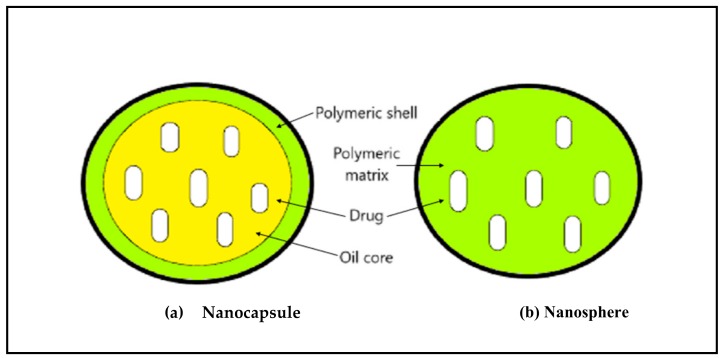
Schematic illustration of (**a**) nanocapsule; and (**b**) nanosphere.

This review focuses on the application of polymeric nanoparticles for the delivery of anti-cancer drugs. These have a size between 10 to 1000 nm and are made up of polymers and copolymers protecting a drug, either encapsulated within particle or adsorbed on the surface or chemically linked to the surface, efficiently [28,29,30]. In 2002, Discher and Eisenberg described the structure of polymeric nanoparticles [31]. They state that it possesses a core-shell structure with the interior consisting of a polymeric matrix containing hydrophobic drug and the surface is made up of hydrophilic polymer e.g., PEG, PVP which provides stearic stability, reduces immunogenicity and phagocytosis of nanoparticles by reticuloendothelial system [32]. In another study, Alonso’s group showed that coating PLA nanoparticles with PEG enhanced their residence time in the GI fluids by protecting from enzymatic degradation [33]. Given these points, polymeric nanoparticles can exist as (1) nanocapsules; or (2) nanospheres (Figure 3).

1. *Nanocapsules:* They have an oily core and a polymeric outer surface. The drug can be adsorbed on the surface or encapsulated in the core.
2. *Nanospheres:* The core and outer surface are made up of polymeric material and the drug is either retained or adsorbed in this polymeric structure.

These polymeric nanoparticles are biodegradable, as the end products are non-harmful alcohols and other low molecular weight products [28]. Furthermore, these increase the solubility of active drug, provide good pharmacokinetic control, are stable, non-toxic, noninflammatory, non-immunogenic, do not activate neutrophils, and avoid reticuloendothelial clearance [34].

To summarize, the conventional medication has several side effects which can be overcome by using these polymeric nanoparticles. For instance, in ophthalmic administration, the nanoparticle releases drug following zero-kinetic order, increasing ocular bioavailability and reducing the side effects [34].

Overall, polymers used as drug delivery vehicle can be classified as synthetic and natural polymers (Table 1).

Synthetic polymers include *N*-(2-hydroxypropyl)-methacrylamide copolymer, polyethylene glycol, poly-l-glutamic acid, poly (lactic-co-glycolic acid), poly (sebacic acid), poly (acrylic acid), etc [29]. The United States Food and Drug Administration had approved Poly(lactic-co-glycolic acid) and Poly(lactic acid) for use by human. Poly(glycolic acid) was the first synthetic polymer used for conjugate building Doxorubicin loaded *N*-(2-Hydroxypropyl)methacrylamide conjugate (Protein-serine Kinase-1) was tested for its anti-cancerous activity [35]. 

Polymers such as heparin, albumin, chitosan, dextran, gelatin are present naturally and are preferred for the delivery of various active constituents such as DNA, drugs, oligonucleotide and proteins. Paclitaxel loaded albumin nanoparticles have been used for the treatment of metastatic cancer [35]. Natural polymeric nanoparticles are preferred because of their reduced side effects, sustained drug release and increased residence time [34].

## 3. Synthesis of Polymeric Nanoparticles

### 3.1. Solvent Evaporation Method

**Figure 4:**
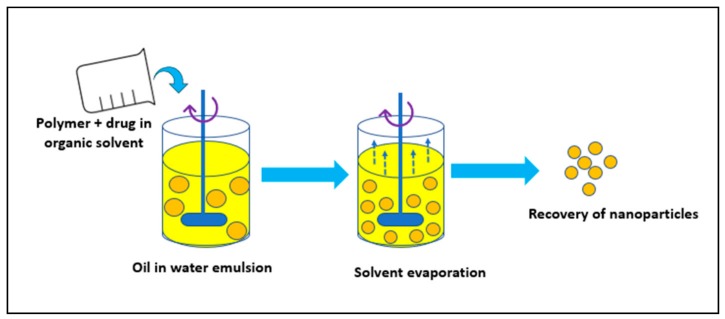
Solvent evaporation method.

It involves addition of the polymer in an organic solvent like chloroform or ethyl acetate followed by dissolution of the drug into polymeric solution forming an oil (O) in water (W) emulsion in the presence of a surfactant like polysorbate-80 or poloxamer-188. Using various physical methods, like increasing the temperature, pressure or by continuous stirring, the organic solvent is evaporated (Figure 4). Given these points, this method is preferred for the synthesis of water-soluble drug-loaded NPs [37]. In short, the procedure is used for laboratory scale synthesis purpose only. 

### 3.2. Spontaneous Emulsification/Solvent Diffusion Method

**Figure 5:**
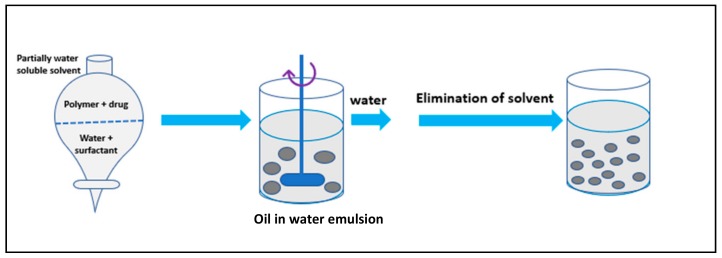
Solvent diffusion method.

This is a modified version of the solvent evaporation method [38,39,40]. Here, the oil phase comprises of water-soluble solvent like methanol or acetone along with the organic solvent like dichloromethane. An interfacial turbulence between the two phases, due to spontaneous diffusion of methanol or acetone (water-soluble solvent), forms nanoparticles (Figure 5).

### 3.3. Salting Out Method

**Figure 6:**
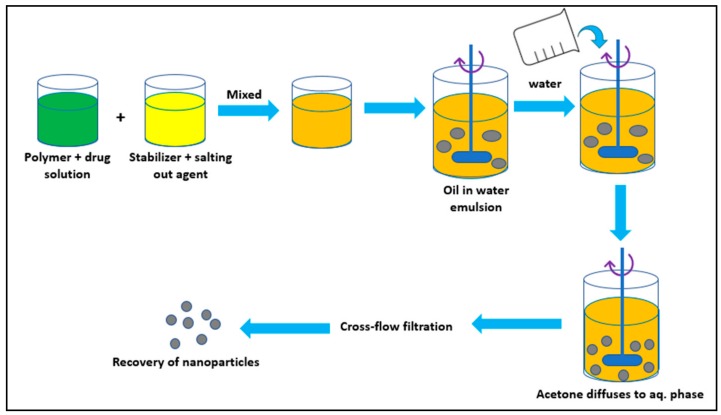
Salting out method.

In brief, to avoid use of organic solvents, this method was developed. It involves dissolution of polymer and drug in a water-soluble solvent like acetone, which is then added to an aqueous solution containing stabilizer like hydroxyethylcellulose or PVP (polyvinylpyrrolidone) and a salting-out agent like magnesium chloride, calcium chloride, and sucrose (Figure 6). This O/W emulsion is then diluted to enhance the diffusion process of acetone into the aqueous phase, forming nanoparticles [41]. In conclusion, it works well for heat sensitive substances as it does not require increase in the temperature [42].

### 3.4. Nanoprecipitation/Solvent Displacement Method

**Figure 7:**
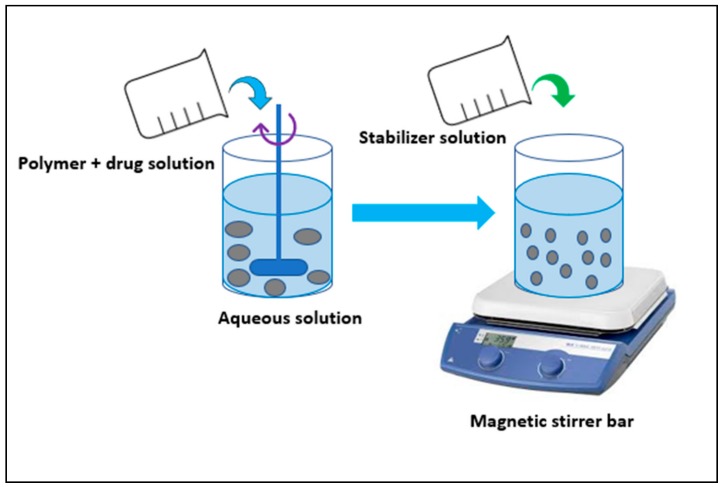
Nanoprecipitation method.

This method is generally preferred for the encapsulation of lipophilic drugs and is not used for water-soluble drugs. It involves addition of polymer and drug into an organic solvent like dichloromethane in the presence or absence of the surfactant. This is then added to an aqueous solution containing stabilizer. An interfacial turbulence between the two phases due to diffusion causes polymer deposition on the interface (Figure 7). Overall, the method is adopted for various materials like PLGA, PLA, Polyvinylmethyl ether and maleic acid (PVM/MA). This method was used for the encapsulation of cyclosporin A, because of 98% entrapment efficiency [26,43,44,45].

### 3.5. Polymerization Methods

Nanoparticles can be synthesised by polymerization of monomers. In a study, Couvreur et al. in 1998 showed the polymerisation process of poly(alkyl cyanoacrylate) [46]. In the first place, the cyanoacrylic monomer is added to a polymerization medium in the presence of a surfactant like polysorbate-20 under vigorous stirring to polymerize ethyl or methyl cyanoacrylate at an ambient temperature. Later, drug is added before the introduction of the monomer or after the polymerization reaction. Usually, ultracentrifugation is used to purify the nanoparticles. Furthermore, to produce stable and high molecular mass NPs, pH (below 3.5), concentration of monomers, stirring speed, type and concentration of the surfactant/stabilizer must be monitored. For instance, a pH above 3 during the polymerization process results in aggregation of the nanoparticles [37].

### 3.6. Nanoparticles Developed from Hydrophilic Polymers

**Figure 8:**
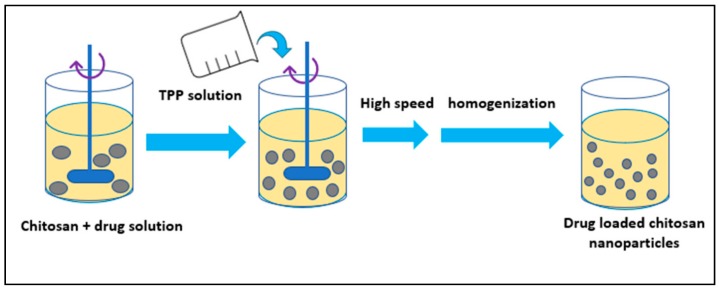
Ion gelation method.

Nanoparticles from hydrophilic polymers like chitosan and gelatin have been synthesized using different methods (Figure 8). In a study, Calvo and coworkers in 1996 developed a method to synthesize chitosan nanoparticles involving ionic gelation using a mixture of two aqueous phases of which first one contained chitosan and EO (ethylene oxide) and the second one contained polyanion sodium-TPP (tripolyphosphate) [47].

In another study, Mao and co-workers in 2001 used complex coacervation method to develop DNA loaded chitosan nanoparticles for oral gene delivery. Additionally, alginate-based nanoparticles were synthesized for oligonucleotides delivery [37,48].

## 4. Chemotherapy and Its Limitations

**Figure 9:**
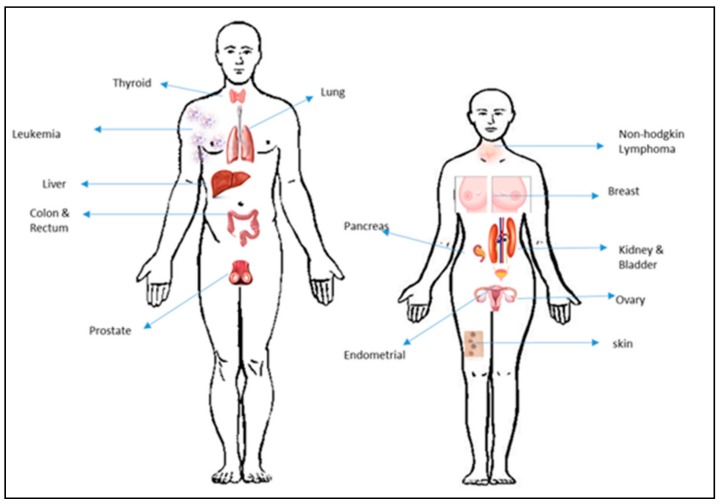
Most common type of cancer in 2018 reported by the American Cancer Society.

Our body is composed of trillions of cells, the most common disease targeting these cells is Cancer. It is a group of diseases where a group of cells start to divide in an abnormal fashion, proliferate without stopping, invading other tissues [49]. Currently, over 100 different types of cancer have been characterized [49,50] they vary mainly in their behavior, location and treatment. For more than a century, cancer has been the focus of research for the scientist around the world. Even though the real reason is still unknown, it is believed to be caused by numerous different factors, such as chemicals, radiation, environmental factors (smoking, obesity, radiation, infections, etc.) acquired and inherited mutations and so on. Based on the Centres for Disease Control and Prevention in the United State, in 2016 1.6 million Americans were diagnosed with cancer (Figure 9). However, remarkable development has taken place in the field of early detection methods for various cancer types, but medical treatment needs attention for the development of efficient and safer medication. Finally, our hope remain in the biological application of nanoparticles, as is a rapidly developing area of nanotechnology that is rising with a new possibility in the diagnosis and treatment of human cancers. Table 2 gives an estimation of the number of new cases and deaths for each cancer type.

In a study, Feng and Chien, in 2003, described chemotherapeutic drugs as active substances that interfere with the activity of a cell by inducing apoptosis or by inhibiting DNA replication [57]. Due to their deleterious effects on rapidly proliferating cells, they have been employed in the treatment of cancer [57,58]. Furthermore, chemotherapeutic drugs, such as Daunorubicin, has been found to show cytotoxic effect on cancer cells and is currently used for the treatment of various tumors such as breast cancer, myeloblastic leukemia and lymphoma [59,60,61]. Related to this, drugs such as Paclitaxel and doxorubicin exert their effect by blocking the cell in the metaphase stage of mitosis [59,62]. Another chemotherapeutic agent, cisplatin, acts by making changes in the cellular DNA triggering apoptosis [59,63]. Doxorubicin also prevents DNA replication by targeting Topoisomerase-DNA complex [59,64].

As has been noted, the main challenge associated with the use of chemotherapeutic agents is their inability to discriminate between healthy and tumor cells [65]. These drugs attack any proliferating cells without differentiating between tumor cells or cells from body such as intestinal epithelial cells or hair cells [58]. Furthermore, side effects associated with doxorubicin such as nausea, fatigue, cardiotoxicity limits its use even after being the best anti-cancer drug available today [59,60]. Considering this, nanocarriers can be employed to overcome some, if not all, limitations associated with the use of these drugs and cytotoxicity against healthy cells is one of them [32,59]. Most notably, the nanocarriers will help in formulation problems such as hydrophobicity of the drug and, they will help in overcoming certain issues such as inappropriate dose and targeted delivery associated with chemotherapy.

## 5. Polymeric Nanoparticles in Cancer Treatment

An important aspect to be overcome by nanocarriers is the drug resistance in cancer cells which occurs over time during chemotherapy treatment. Various mechanisms by which this can be done include:

1 Cellular uptake of drug via endocytosis or receptor-mediated internalization [66,67,68]
2 Polymeric material such as Pluronic block copolymers can be used to inhibit the multidrug resistance proteins [67]
3 Increasing the concentration of drug around the tumor cell [66]
  Both inhibitor and drug can be loaded inside the nanoparticle for synergistic effect [69]

Polymeric nanoparticles are promising candidates in the treatment of cancer due to their biodegradable nature, sustained release of drug, nanosize, biocompatibility, bioactivity, non-toxic nature, long circulation time, non-immunogenicity and ability to hold various active molecules such as drugs, oligonucleotide, peptides, etc. [70,71]. This part of review focuses on the ongoing research in the field of nanomedicine and the use of polymeric nanoparticles for the delivery of anti-cancer agents.

## 6. Poly-d,l-lactide-co-glycolide (PLGA)

PLGA is listed safe by US Food and Drug Administration for human use. It is the most successful candidate for use in nanomedicine due to the formation of biodegradable by products viz., lactic acid and glycolic acid, upon its hydrolysis. The various synthesis method includes solvent evaporation, interfacial deposition, emulsification-diffusion and nanoprecipitation (most commonly used). However, the acidic nature of PLGA makes it less suitable as carrier for drugs and bioactive molecules [71,72,73]. Therefore, this limitation is overcome by blending PLGA with other polymers such as poly(propylenefumarate) [71,74], polyvinylalcohol [71,73,75] chitosan etc. In this context, few of the PLGA based nanosystems as carriers for anti-cancer drugs are as follows.

### 6.1. Cisplatin

Cisplatin is a potent anti-cancer drug that interferes with the cell division. However, its use is limited due to its cytotoxicity against healthy cells [71,76]. Therefore, targeted delivery of this drug to tumor cells is increased by encapsulating it within PEG-PLGA nanosystem. 

### 6.2. Curcumin

Curcumin is effective against prostate cancer, but the hydrophobicity of the drug limits its use. In a study by Mukherjee and Vishwanatha in 2012, they developed curcumin loaded PLGA nanospheres using emulsion solvent evaporation method with increased anti-cancer efficacy [77].

### 6.3. Docetaxel

Docetaxel is specific for cancer cells with folate receptors. Hence, its efficiency is increased by loading it inside PEG-PLGA nanoparticles synthesized using emulsion solvent diffusion [78]. Comparatively, it leads to enhanced cellular uptake by cancer cells.

### 6.4. 9-Nitrocamptothecin (9-NC)

9-NC is a promising anti-cancer agent that targets the topoisomerase-1 enzyme. But, due to its pH dependent fast and reversible hydrolysis its use is limited. Hence, the biological activity of this drug is enhanced by loading it inside PLGA nanoparticles by nanoprecipitation method [71,76].

### 6.5. Paclitaxel

Paclitaxel acts by interfering with the cell dynamics by promoting polymerization of tubulin and hence, initiating cell death. It is important to mention that it shows anti-cancer activity against breast, colon and ovarian cancer [71]. Given these points, the poor solubility issue is overcome by encapsulating the drug in a PLGA-Vit E-TPGS complex which is synthesized using solvent evaporation or extraction method.

### 6.6. Rose Bengal

Rose Bengal is specific for tumor cells and gets localized in the lysosomes. Therefore, it is used for the treatment of melanoma cancer [79]. However, the efficiency of the drug is enhanced by loading it inside PLGA nanoparticles by interfacial deposition method. 

## 7. Poly-ε-caprolactone (PCL)

PCL has received great attention worldwide for use in nanomedicine because of the hydrolysis of its ester bond at physiological pH making it biodegradable. It is synthesized by nanoprecipitation, solvent displacement and solvent evaporation method [71]. Some of the anti-cancer drug loaded PCL based nanosystems are as follows.

### 7.1. Docetaxel

Docetaxel has anti-tumor effect. Hence, the surface modification of PCL nanoparticles with Me PEG, synthesized using nanoprecipitation method, allows effective killing of B 16 cells [80].

### 7.2. Vinblastine

Vinblastine is effective against breast cancer. Therefore, the Vinblastine loaded PCL nanoparticles synthesized using emulsion method allows efficient uptake of nanomedicine by the cancer cells with a slow drug release profile [81].

### 7.3. Tamoxifen

Tamoxifen works by binding to the estrogen receptors on the target cells and inhibiting estrogen effect. It is an anti-estrogen drug and competes with estrogen for the receptor binding on the breast and other tissues. Hence, it prevents proliferation of pre-cancerous cells by arresting the cell in G_0_ and G_1_ phase of the cell cycle [81]. Therefore, the tamoxifen encapsulated PCL nanoparticles are synthesized using solvent displacement method allowing enhanced circulation time and targeted delivery to the tumor site with increased drug accumulation level [82].

### 7.4. Taxol

Taxol has tumor growth inhibition activity. The extremely lipophilic characteristic of taxol could be overcome by encapsulating the drug in mPEG-PCl nanoparticles with enhanced anti-cancer activity due to higher drug loading [83].

## 8. Gelatin

Gelatin is a natural polymer with polyampholyte nature along with hydrophilic moiety. It is used for the sustained/controlled drug release due to its biodegradable, bioactive, biocompatibility, non-toxic, mechanical and thermal properties. It is synthesized using emulsion, coacervation/desolvation method [84,85].

### Paclitaxel

Paclitaxel has anti-cancer activity against human-bladder transitional cancer cell. The paclitaxel encapsulated gelatin nanoparticles synthesized using desolvation method have increased anti-cancer efficacy compared to the free drug. The increased water solubility of the nanoparticle due to its amorphous nature allows rapid drug release at the target site [86].

## 9. Poly-alkyl-cyano-acrylates (PAC)

PAC have adverse effects on the central nervous system as the by-products are toxic and hence, PAC is not regarded as safe for human use, overshadowing its biodegradable and biocompatible nature. It is synthesized using emulsion, interfacial polymerization and nanoprecipitation method for use in nanomedicine [87,88]. An example of PAC loaded nanosystem is Ftorafur drug.

### Ftorafur

Ftorafur is a mixture of tegafur and uracil. Tegafur when taken up by cancer cell breaks down into 5-FU subsequently killing it. Uracil on the other hand increases the level of 5-FU inside the cell, killing the cancer cell. Tegafur and uracil have synergistic effect. The ftorafur encapsulated PE-2-CA and PBC nanospheres have wide range of anti-tumor activity allowing rapid drug release at the target site [89].

## 10. Targeted Delivery of Nanoparticles

To begin with, two basic requirements must be met by the anti-cancer drugs for effective treatment of cancer [15]. These include:

1 Reaching the site of action after crossing all the biological barriers while retaining their activity with minimum loss of volume.
2 Attacking the tumor cells with minimal cytotoxic effect on the healthy neighboring cells or the tissues.

**Figure 10:**
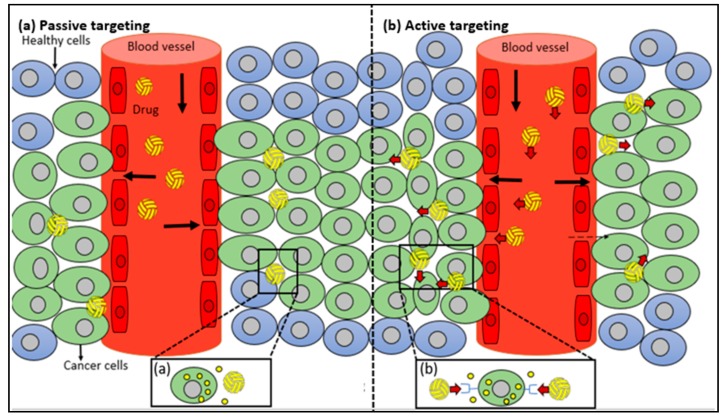
Schematic illustration of (**a**) Passive targeting; (**b**) Active targeting.

Nanoparticles fulfil both the requirements and hence act as a good drug carrier system. Types of targeting are as follows (Figure 10).

### 10.1. Passive Targeting

Increased permeability: The tumor cells and inflamed tissues have leaky microvasculature with numerous pores in the range of 380 and 780 nm [35], unlike healthy cells. This happens due to the increased demand of oxygen and nutrients by the hyper-proliferating cancer cells demanding neo-vascularization, called Enhanced Permeability and Retention effect (EPR) [90].

Tumor microenvironment: The tumor cells have an acidic environment as they use glycolysis pathway for meeting extra-energy demand due to increased metabolism [91].

For passive targeting, the nanoparticles must be able to accumulate in the tumor interstitium. Furthermore, the circulating life in blood should be long so that they have multiple chances to pass the target site. This can be achieved by coating the nanoparticles with surfactants such as PEG which is chemically inert, has low immunogenicity and antigenicity [92]. However, this may cause the passive distribution of drug to multiple sites. But, in some cases this situation may be beneficial too [15].

### 10.2. Active Targeting

Tumor and inflamed tissues not only have leaky vasculature but also have overexpression of certain epitopes and receptors that can be targeted. Once the nanocarriers have extravasated the tissue, the ligands on the surface of these nanocarriers can bind to the epitopes and receptors overexpressed on cancer cells allowing cellular uptake by receptor-mediated endocytosis. This is referred to as “active mode of targeting enhancing availability” of drug with poor permeability requiring intracellular site of action for bioactivity [93]. This can enhance the biodistribution of nanomedicine [94]. This method of targeting has been employed to delivery drug to drug resistant cancer cells. Key factor to be kept in mind while choosing ligands is its ability to activate Mononuclear Phagocytic system (MPS) (Table 3).

The active mode of targeting has several advantages over ligand-drug conjugates which includes:

1 High concentration of drug can be transported to the site of action.
2 The activity of the drug can be affected by conjugating ligand with the drug which is not the case in active targeting using ligand tagged nanocarriers.
3 Numerous ligands can be attached on the surface of nanocarriers increasing chance to pass the target site.

## 11. Polymeric Nanoparticles Related Toxicity Issues

Nowadays nanomaterials are of keen interest to the scientist in the biomedical field due to their wide range of applications in diagnosis, drug delivery, and development of human organs [103,104]. However, the biggest concern is the safety associated with the use of these NPs. The bare and small size of these particles have higher toxicity than modified and bulk materials, respectively. For instance, spherical nanoparticles have less toxicity than rod shaped nanoparticles due to their ability to trigger an immune response in the body. The toxicity of these materials can be reduced by chemical approaches such as surface treatment, functionalization, and composite formation [105]. There are many factors that could conceivably influence material’s toxicity, these includes surface chemistry, roughness, surface energy (hydrophobicity/hydrophilicity), level of degradation, products and release of by-products, particle size, oxidative stress functions, crystallinity, concentration, coating, and the longevity of particles [103]. The toxicity may vary in severity depending on the mode of administration and site of release, as a result, to preserve clinical purpose, information on toxicity is exhibited using a system-based approach focusing on lung, dermal, liver, and nervous system targets.

The interaction between NPs and living systems are not yet fully understood. The problem arises with the particles ability to bind and interact with biological material which can alter their surface characteristics, depending on the environment they are delivered to. Scientific knowledge about NPs cell-interaction mechanisms has been indicating that cells readily take up NPs via active or passive mechanisms. 

At the end, even though these nanoparticles are a huge advancement in drug designing, it is better to have more understanding of the effects that they may have on human health before heading towards their clinical usage. They need to be evaluated on a particle-by-particle basis and must undergo better characterization strategy such as absorption, distribution, metabolism and excretion (ADME) tests and physicochemical, and toxicological characterization, involving both in vitro tests and in vivo animal studies. 

## 12. Conclusions and Outlook

Nanoparticles used for drug delivery provide many advantages in medicine because of enhanced drug-therapeutic efficiency. They enter the body and bind to biological tissue and cells. After entry, they tend to dissolve and enter the biological environment, surrounded by proteins, high ionic strength, and low pH [106]. Nanoparticles interacts with the skin, the gastrointestinal tract, and the respiratory tract, and many others compartments that act as barriers to this nanosized material in the organism. A new development of a multifunctional Nanosystems have combined, different functions in a single nanoparticle, such as biocompatibility, biostability and biodistribution. 

The polymeric nanoparticle is one of the most preferred nanoparticulate delivery systems in medicine and include synthetic polymers and natural polymers [107]. Polymeric nanoparticles, and its architecture, composition, stability, water solubility make them effective candidate for use in drug delivery. Based on several studies, it has been demonstrated that a polymeric carrier has controlled/sustained drug release, bioavailability and biodistribution in the body. Based on their polymeric characteristic, these nanoparticles have significance in medical field [108].

One of the best characteristics of polymeric nanoparticles is that they provide a buoyed-up release of encapsulated drugs, help to protect drugs from the body’s enzymatic, acidic, and degradation conditions, provide targeting capabilities from a tendency for passive accumulation in tumors, and display adjuvant characteristics. These and other properties make this type of nanoparticle a suitable mechanism to prevent cancer attacks. Priscilla B et al. 2017, used biocompatible Polymeric Nanoparticles derived from Castor Oil Derivatives for biomedical applications because of their biodegradability and biocompatibility [109].

Finally, multifunctional nanoparticles can facilitate visualization of malignant cells (using in vivo imaging), target them (through active targeting ligand) eventually killing the cancer cells without harming neighboring healthy cells via active targeting and controlled release of drug.

## Figures and Tables

**Table 1:** Synthetic and natural biodegradable polymers used as drug delivery vehicles adapted from [36].

Synthetic Biodegradable Polymers		Natural Biodegradable Polymers	
Polyesters	Polyoxalates	Starch	Chitosan
Polyorthoesters	Polyiminocarbonates	Hyaluronic acid	Dextran
Polyanhydrides	Polyurethanes	Heparin	
Polydioxanones	Polyphosphazenes	Gelatin	
Poly(a-cyanoacrylates)		Albumin	

**Table 2:** List of cancers, their cause, common types, and estimated deaths.

Most Prevalent Cancers	Cause	Most Common Type	Estimated Death	References
Bladder	Smoking, Exposition to certain chemicals, chronic bladder infections, Abnormal cell growth in the muscular sac that stores urine, urothelium, infection with *Schistosoma haematobium*	Urothelial carcinoma, squamous cell carcinoma, adenocarcinoma, Superficial bladder cancer, Invasive bladder cancer	17,240	[51,52]
Breast (Men and Female)	Malignant tumor in the breast, gene mutation, family history	Ductal carcinoma in situ (DCIS), Invasive Ductal Carcinoma (IDC), Mammogram, Lumpectomy, Mastectomy	268,670	[51]
Colon and Rectal	Genetic mutation, an inherited or acquire mutation to the APC gene.	More than 95% of colon cancer can be classified as adenocarcinomas.	50,630	[51]
Endometrial	Increasing age, unopposed estrogen therapy, late menopause, tamoxifen therapy, nulliparity, infertility or failure to ovulate, obesity, hypertension, diabetes, and HNPCC.	Adenocarcinoma, Carcinosarcoma, Squamous cell carcinoma, Undifferentiated carcinoma, Small cell carcinoma, Transitional carcinoma	11,350	[53]
Kidney (Renal Cell and Renal Pelvis)	Smoking, obesity, Workplace exposures, Family history of kidney cancer, High blood pressure, Certain medicines	Renal cell carcinoma (RCC), Clear cell renal cell carcinoma, Papillary renal cell carcinoma, Chromophobe renal cell carcinoma, transitional cell carcinomas, Wilms tumors, and renal sarcomas.	14,970	[51,54]
Leukemia (All Types)	DNA of immature blood cells, mainly white cells, becomes damaged.	Acute lymphoblastic leukemia, Acute myeloid leukemia, Chronic lymphocytic leukemia (CLL), Chronic myeloid leukemia (CML), Chronic myelomonocytic leukemia (CMML), Leukemia in children	24,370	[51]
Liver and Intrahepatic Bile Duct	Alcohol, age, smoking, genetic, hepatitis, obesity, cirrhosis, gender	Hepatocellular carcinoma, Hepatoblastoma, Hepatocellular carcinoma	30,200	[51]
Lung (Including Bronchus)	Smoking tobacco, second hand smoke, genetic undergoing radiation therapy and environmental exposure	Small Cell Lung Cancer (SCLC) Non-Small Cell Lung Cancer (NSCLC)	154,050	[51,55]
Skin	Exposure to Ultraviolet (UV) light, tanning beds or sunlamps.	Melanoma, Basal, Squamous and Merkel Cell Carcinoma, Epidermoid cysts	9320	[51,55]
Non-Hodgkin Lymphoma	Infection-fighting cells of the immune system, called lymphocytes, immune deficiency.	Hodgkin lymphoma and non-Hodgkin lymphoma (NHL), Skin lymphoma, pediatric lymphoma, AIDS-related lymphoma, Waldenstrom macroglobulinemia (WM)	19,910	[51]
Pancreatic	DNA mutations, Diabetes, smoking, pancreatitis, smoking, Obesity	Exocrine cancers, Pancreatic adenocarcinoma, endocrine	44,330	[51]
Prostate	Oncogenes change or mutation in the DNA, Age, growth of abnormal cells, which may invader healthy cells in the body.	Acinar, ductal adenocarcinoma, ductal, urothelial, squamous cell cancer, Small all prostate cancer	29,430	[56]
Thyroid	Radiation, low iodine consumptions, family history, gender, age, hereditary conditions, DNA mutations	papillary, follicular, medullary, and anaplastic thyroid cancer.	2060	[51]

**Table 3:** Example of some ligands used in active drug targeting adapted from [95].

Targeting Ligands	Targets	References
Aptamers	Antibodies, cell surface receptors, enzymes, small organic molecules, peptides, proteins	[96,97]
Folate	Folate receptor	[98]
Gelatinase inhibitor peptide CTTHWGFTLC	Matrix Metalloprotease-2 and Matrix Metalloprotease-9 gelatinase	[99]
Luteinizing hormone-releasing hormone	Luteinizing hormone-releasing hormone receptor	[100,101]
RGD peptide	Integrin	[102]
